# The name Ixodes dammini epidemiologically justified.

**DOI:** 10.3201/eid0401.980126

**Published:** 1998

**Authors:** S R Telford


throughout the city, although many patients came from the same area.

A seroepidemiologic study to determine possible new sources of infection (e.g., dogs, cats) and estimate rates of seropositivity in cattle and sheep and a case-control study on new cases are being conducted.

**F. Pfaff,* A. François,* D. Hommel,† I. Jeanne,* J. Margery,‡ G. Guillot,† Y. Couratte-Arnaude,‡ A. Hulin,† and A. Talarmin***
*Pasteur Institute of French Guiana, Cayenne, French Guiana; †Cayenne Hospital, Cayenne, French Guiana; and ‡Army Health Care Service, Cayenne, French Guiana

### Reference

1. Floch H. La pathologie vétérinaire en Guyane française (les affections des porcins, des caprins et des ovins). Revue Elev Méd Vét Pays Trop 1955;8:11-3.

### *Ixodes dammini:* A Junior Synonym for *Ixodes scapularis*

**To the Editor:** The authors of "A new tick-borne encephalitis-like virus infecting New England deer ticks, *Ixodes dammini*" (1) provide useful information regarding a possibly new tick-borne encephalitis-like virus. However, the use of the name *Ixodes dammini* is not accurate for describing this species. *I. dammini* (Spielman, Clifford, Piesman, and Corwin) was synonymized with *Ixodes scapularis* (Say) in 1993 by Oliver et al. (2) and was redescribed in 1996 (3) to reduce confusion regarding identification. Keirans and colleagues summarize a wide array of rigorous studies involving hybridization, assortative mating, isozymes, and morphometrics, all of which provide evidence supporting the synonymization of the two tick species (3).

The synonymization of *I. dammini* with *I. scapularis* has been widely accepted. "*I. scapularis* (= *I. dammini*)" is still often used, but the use of *I. scapularis* as the sole nomen for this species is becoming more common (4). Oliver et al. (2) have established *I. dammini* as a junior subjective synonym of *I. scapularis.* If scientifically rigorous evidence exists justifying the reestablishment of the species name *I. dammini,* it must be published according to proper procedure. The proper nomenclature of any species, let alone one of such widespread notoriety and public health importance, is too important to be relegated to a footnote. Until such evidence is presented, the continued misuse of *I. dammini* serves only to confuse health-care providers, public health professionals, and lay persons.

On a secondary matter, on page 167 of the dispatch, the authors state that "*I.* (Pholeoixodes) *cookei* is a one-host tick that is only distantly related to *I. dammini* and only rarely feeds on humans or mice" (1). *I. cookei* is a three-host tick (D.E. Sonenshine, pers. comm.), as are all the members of the genus Ixodes.

**Martin Sanders**
Maryland Department of Health and Mental Hygiene, Baltimore, Maryland, USA

### References

1. Telford SR III, Armstrong PM, Katavolos P, Foppa I, Garcia ASO, Wilson ML, Spielman A. A new tick-borne encephalitis-like virus infecting New England deer ticks, *Ixodes dammini*. Emerg Infect Dis 1997;3:165-70.
2. Oliver JH, Owsley MR, Hutcheson AM, James C, Chen W, Irby S, et al. Conspecificity of the ticks *Ixodes scapularis* and *Ixodes dammini* (Acari: Ixodidae). J Med Entomol 1993;30:54-63.
3. Keirans JE, Hutcheson HJ, Durden LA, Klompen JSH. *Ixodes scapularis* (Acari: Ixodidae): redescription of all active stages, distribution, hosts, geographical variation, and medical and veterinary importance. J Med Entomol 1996;33:297-318.
4. Centers for Disease Control and Prevention. Lyme disease—United States, 1996. MMWR Morb Mortal Wkly Rep 1997;46:531-5.

### The Name *Ixodes dammini* Epidemiologically Justified

**To the Editor:** Although a large body of evidence has been interpreted as supporting conspecificity of the deer tick (*Ixodes dammini*) and the blacklegged tick (*Ixodes scapularis*), according to Chapter VI, Article 23 L of the International Code of Zoological Nomenclature (1), "A name that has been treated as a junior synonym may be used as the valid name of a taxon by an author who considers the synonymy to be erroneous...."

Current use of *I. scapularis* to refer to the vector of Lyme disease obscures important epidemiologic issues. One of the reasons for "sinking" *I. dammini* was to make it easier to diagnose Lyme disease in areas where the disease was thought to be nonendemic: "The belief that *I. dammini* does not occur south of Maryland and that *I. scapularis* is a separate and distinct species yet unproven as a natural vector of Lyme disease has caused delays in Lyme disease surveillance in the South. The general attitude among physicians and veterinarians has been that Lyme disease is not a problem in that area, although patients present clinical symptoms of it" (2). Recognizing and reporting Lyme disease in southern and southcentral states should not, however, depend on whether the two ticks are conspecific. Only peer-reviewed descriptions of human cases of Lyme disease, with appropriate documentation of the diagnoses, should be accepted as evidence. Few such reports exist, and the evidence does not convincingly support a conclusion that Lyme disease exists as an epidemic zoonosis in southern states (3). This is not to say that residents outside the well-established eastern United States zoonotic sites (the Northeast and upper Midwest) do not have symptoms that fit one or more aspects of the current Centers for Disease Control and Prevention/Council of State and Territorial Epidemiologists/Association of State and Territorial Public Health Laboratory Directors surveillance definition for Lyme disease. Lyme disease-like infections, mainly manifesting as erythema migrans and strongly associated with Lone Star tick (*Amblyomma americanum*) bites are commonly seen in southern and southcentral states, but *Borrelia burgdorferi* does not seem to be the etiologic agent (4).

Enzootic transmission of Lyme disease spirochetes among rodents and ticks had been documented in southern and southcentral states by the late 1980s (5-7). The question, however, is whether there is frequent zoonotic transmission. There are widespread southern U.S. enzootic cycles of *Trypanosoma cruzi*, but few autochthonous human Chagas disease cases seem to occur because the vectors (such as *Triatoma sanguisuga*) have behavioral traits that reduce their capacity to serve as zoonotic vectors (8). Nymphal *I. scapularis* apparently do not frequently bite humans (7,9), although adult ticks do. The major feature of Lyme disease epidemiology in the Northeast and in the upper Midwest, however, is transmission by nymphal *I. dammini* (10).

Whether the predilection of nymphal *I. dammini* to feed on humans is environmentally determined or is a heritable trait with undescribed genetic markers remains unexplored. Particular mitochondrial DNA haplotypes seem to be more characteristic of *I. dammini* (11,12), and the use of such typing methods may enhance future analyses of the vectorial capacity of these ticks. For example, one might test the hypothesis that nymphal ticks removed from residents of sites in coastal North Carolina through Georgia, where both kinds of ticks have been collected, represent only *I. dammini*. But, if it is "widely accepted" that no differences exist between the two ticks, such studies may never be done. Similarly, many may wrongly assume that Lyme disease, human babesiosis, and human granulocytic ehrlichiosis are, or will become, epidemic throughout virtually all of the eastern United States. An equally likely scenario is that these zoonoses may never become public health problems for more southerly states. For the moment, then, distinguishing tick populations that frequently bite humans from those that rarely do seems to be a rational use of nomenclature, particularly for public health officials.

Dr. Sanders is correct in pointing out that all *Ixodes* spp. are "three-host" ticks, although my intent in using the term "one-host" was to indicate that all stages of *I. cookei* tend to feed on the same kind of animal (sometimes a single animal, within burrows), usually woodchucks, skunks, or raccoons. I regret the confusion from my use of the acarologic term in a descriptive context.

**Sam R. Telford III**
Harvard School of Public Health,
Boston, Massachusetts, USA

### References

1. International code of zoological nomenclature. 3rd ed. London: International Trust for Zoological Nomenclature; 1985.
2. Oliver JH Jr, Owsley MR, Hutcheson HJ, James AM, Chen C, Irby WS, et al. Conspecificity of the ticks *Ixodes scapularis* and *I. dammini* (Acari: Ixodidae). J Med Entomol 1993;30:54-63.
3. Barbour AG. Does Lyme disease occur in the South? A survey of emerging tickborne infections in the region. Am J Med Sci 1996;311:34-40.
4. Campbell GL, Paul WS, Schriefer ME, Craven RB, Robbins KE, Dennis DT, et al. Epidemiologic and diagnostic studies of patients with suspected early Lyme disease, Missouri, 1990-1993. J Infect Dis 1995;172:470-80.
5. Levine JF, Apperson CS, Nicholson WL. The occurrence of spirochetes in ixodid ticks in North Carolina. Journal of Entomological Science1989;24:594-602.
6. Kocan AA, Mukolwe SW, Murphy GL, Barker RW, Kocan KM. Isolation of *Borrelia burgdorferi* (Spirochaetales: Spirochaetaceae) from *Ixodes scapularis* and *Dermacentor albopictus* ticks (Acari: Ixodidae) in Oklahoma. J Med Entomol 1992;29:630-3.

7. Luckhart S, Mullen GR, Wright JC. Etiologic agent of Lyme disease, *Borrelia burgdorferi*, detected in ticks (Acari: Ixodidae) collected at a focus in Alabama. J Med Entomol 1991;28:652-7.
8. Pung OJ, Banks CW, Jones DN, Krissinger MW. *Trypanosoma cruzi* in wild raccoons, opossums, and triatomine bugs in southeast Georgia, USA. J Parasitol 1995;81:324-6.
9. Lavender DR, Oliver JH Jr. Ticks (Acari:Ixodidae) in Bulloch County, Georgia. J Med Entomol 1996;33:224-31.
10. Spielman A, Wilson ML, Levine JF, Piesman J. Ecology of *Ixodes dammini* borne human babesiosis and Lyme disease. Annu Rev Entomol 1985;30:439-60.
11. Rich SM, Caporale DA, Telford SR III, Kocher TD, Hartl DL, Spielman A, et al. Distribution of the *Ixodes ricinus*-like ticks of eastern North America. Proc Natl Acad Sci U S A 1995;92:6284-8.
12. Norris DE, Klompen JSH, Keirans JE, Black WC IV. Population genetics of *Ixodes scapularis* (Acari:Ixodidae) based on mitochondrial 16S and 12S genes. J Med Entomol 1996;33:78-89.

### Ebola/Athens Revisited

**To the Editor:** After our hypothesis that the plague of Athens (430 B.C.–425 B.C.) could have been caused by Ebola virus was published in this journal (1996;2:155-6), it was brought to our attention that this hypothesis had been previously entertained.

Gayle D. Scarrow had published a paper entitled “The Athenian Plague: A Possible Diagnosis” in The Ancient History Bulletin 2.1 (1988). Unfortunately, this had not come to our attention in our literature search, and therefore we assumed that we were the first to recognize the possibility. Clearly, Ms. Scarrow deserves credit for suggesting this first. Her arguments are compelling, even without the support of more recently available information and the observations advanced in our publication.

We believe an evolving knowledge base (e.g., the information about the Côte d’Ivoire outbreak where a protracted epidemic has been meticulously documented) will serve to enhance the credibility of the Ebola/Athens hypothesis.

**P.E. Olson,* A.S. Benenson,† and E.N. Genovese†**
*U.S. Navy Balboa Hospital, San Diego, California, USA; †San Diego State University, San Diego, California, USA

